# Delivery of stem cells to the brain: potential for treatment of neurodegenerative disease

**DOI:** 10.1186/2050-5736-3-S1-O17

**Published:** 2015-06-30

**Authors:** Alison Burgess, Isabelle Aubert, Kullervo Hynynen

**Affiliations:** 1 Sunnybrook Research Institute, Toronto, Ontario, Canada

## Background/introduction

Stem cell therapy is a promising strategy to treat neurodegenerative diseases, including Alzheimer’s and Parkinson’s diseases. However, for stem cell therapy to be considered for clinical use, new delivery methods need to be developed. We have used focused ultrasound (FUS) as a method to induce transient opening of the blood-brain barrier (BBB) to promote the passage of therapeutic stem cells into the brain.

## Methods

As a proof of principle study to demonstrate the application of this therapeutic strategy for Parkinson’s disease, we targeted the striatum using magnetic resonance imaging (MRI) for BBB opening. Definity microbubble contrast agent was injected intravenously at the onset of FUS application using a 558 kHz transducer (~0.3 MPa estimated *in situ* pressure, 10 ms bursts, 1 Hz pulse repetition frequency, 120 s total exposure duration).

## Results and conclusions

During FUS application, neural stem cells expressing green-fluorescent protein (GFP) and labeled with superparamagnetic iron oxide (SPIO) were injected into the carotid artery. Follow up contrast enhanced MR imaging confirmed the opening of the BBB and at 24 hrs, MRI was used to detect the SPIO labeled stem cells in the region of interest. After sacrifice, GFP expressing cells were present in the targeted regions of the brain and expressed a neuronal phenotype indicative of their survival post-delivery through the BBB. Histological analysis showed some red blood cell extravasation but there was no evidence of brain tissue damage due to FUS treatment. Together, these data suggest that FUS is an efficient method for delivery of stem cells to the brain and may be the key to reducing the risks of cell transplantation helping to move stem cell therapy forward.

